# Glucagon-Like Peptide-1 Analog, Liraglutide, Delays Onset of Experimental Autoimmune Encephalitis in Lewis Rats

**DOI:** 10.3389/fphar.2016.00433

**Published:** 2016-11-18

**Authors:** Brian DellaValle, Gitte S. Brix, Birgitte Brock, Michael Gejl, Anne M. Landau, Arne Møller, Jørgen Rungby, Agnete Larsen

**Affiliations:** ^1^Department of Biomedicine/Pharmacology, Aarhus UniversityAarhus, Denmark; ^2^Centre of Medical Parasitology, Department of Clinical Microbiology, Copenhagen University HospitalCopenhagen, Denmark; ^3^Department of Clinical Biochemistry and Department of Clinical Medicine, Aarhus University Hospital, Aarhus UniversityAarhus, Denmark; ^4^Department of Nuclear Medicine and PET Center, Aarhus UniversityAarhus, Denmark; ^5^Centre For Functionally Integrative Neuroscience, Aarhus UniversityAarhus, Denmark; ^6^Department of Endocrinology, Bispebjerg University HospitalCopenhagen, Denmark

**Keywords:** GLP-1, EAE, multiple sclerosis, liraglutide, MS, MnSOD, APP

## Abstract

**Introduction:** Recent findings indicate that metabolic disturbances are involved in multiple sclerosis (MS) pathology and influence the susceptibility to treatment, directing attention toward anti-diabetic drugs such as metformin and pioglitazone. Liraglutide, a drug of the glucagon-like peptide-1 (GLP-1) family, is also anti-diabetic and weight-reducing and is, moreover, directly neuroprotective and anti-inflammatory in a broad spectrum of experimental models of brain disease. In this study we investigate the potential for this FDA-approved drug, liraglutide, as a treatment for MS by utilizing the experimental model, experimental autoimmune encephalitis (EAE).

**Methods:** EAE was induced in 30 female Lewis rats that subsequently received twice-daily liraglutide (200 μg/kg s.c.) or saline. Healthy controls were included (saline, *n* = 6, liraglutide, *n* = 7). Clinical score and weight were assessed daily by blinded observers. Animals were killed at peak disease severity (day 11) or if exceeding humane endpoint (clinical score ≥4). Protein levels of manganese superoxide dismutase (MnSOD), amyloid precursor protein (APP), and glial fibrillary acidic protein (GFAP) were determined.

**Results:** Liraglutide treatment delayed disease onset (group clinical score significantly >0) by 2 days and markedly reduced disease severity (median clinical score 2 vs. 5; *p* = 0.0003). Fourteen of 15 (93%) of vehicle-treated rats reached the humane endpoint (clinical score ≥4) by day 11 compared to 5 of 15 (33%) of liraglutide-treated rats (*p* = 0.0004). Liraglutide substantially increased the mitochondrial antioxidant MnSOD (*p* < 0.01) and reduced the neurodegenerative marker APP (*p* = 0.036) in the brain. GFAP levels were not significantly changed with drug treatment (*p* = 0.09).

**Conclusion:** We demonstrate, for the first time, that liraglutide treatment delays onset of EAE in Lewis rats and is associated with improved protective capacity against oxidative stress. These data suggest GLP-1 receptor agonists should be investigated further as a potential therapy for MS.

## Introduction

Current multiple sclerosis (MS) treatments are non-curative, side-effect prone, and expensive, highlighting the need for expanded treatment options for patients. Newly diagnosed MS patients exhibit hyperinsulinemia and decreased insulin sensitivity (Penesova et al., 2015) suggesting that obesity is a potential risk factor for MS (Palavra et al., 2016). Treating underlying metabolic syndrome with classic anti-diabetic drugs such as metformin and pioglitazone ameliorates metabolic disturbances, reduces MRI-evident lesion frequency and dampens T-cell pro-inflammatory response in MS patients (Negrotto et al., 2016). Metformin also reduces disease severity and pro-inflammatory response in an experimental model of MS (Nath et al., 2009; Sun et al., 2016). Recently, obese MS patients have been shown to have a less pronounced response to interferon treatment (Kvistad et al., 2015) underlining the need for further investigation of metabolic disturbances and pharmacological targets for treating MS through improved metabolic control.

The glucagon-like peptide-1 (GLP-1) class of anti-diabetic drugs improve metabolic control and moreover, have a direct neuroprotective potential in humans (Gejl et al., 2014; Candeias et al., 2015). In this study, we were interested in the GLP-1 receptor agonist, liraglutide: a long-acting GLP-1 analog designed to extend the half-life of GLP-1 receptor activation (Knudsen et al., 2000) that can cross the blood-brain barrier (Hunter and Hölscher, 2012). Indeed, we have previously shown that liraglutide stabilizes cerebral glucose consumption in healthy subjects (Gejl et al., 2014) and Alzheimer's patients (Gejl et al., 2016) and reduces lesion size, cell death and oxidative damage in experimental traumatic brain injury (DellaValle et al., 2014). Liraglutide treatment is associated with increased the levels of numerous neuroprotective proteins associated with mitochondrial function (DellaValle et al., 2014) and moreover; GLP-1 receptor activation has been shown to be anti-inflammatory (Parthsarathy and Holscher, 2013; DellaValle et al., 2014; Candeias et al., 2015).

Here we investigate the potential of liraglutide as a candidate MS therapy by assessing pre-clinical efficacy in an active, monophasic rat model of experimental autoimmune encephalitis (EAE). This model is characterized by an aggressive onset and we were primarily interested in the effect of liraglutide on the induction phase of EAE.

## Materials and methods

Female Lewis rats (Charles River, Germany) aged 11–12 weeks, weighing ~210 g were housed under standard conditions. Studies were conducted to minimize suffering and were approved by the Danish Animal Inspectorate (2015-15-0201-00647). Weight was monitored daily throughout the experiment.

### EAE induction

EAE Emulsion: 100 μL complete Freund's adjuvant (CFA; BD 263810, Denmark (DK)), 200 μg *Mycobacterium tuberculosis* H37Ra (MT; BD, 231141, DK), 100 μg guinea pig myelin basic protein (MBP; Sigma-Aldrich, DK, M2295), and 100 μL 0.9% saline.

EAE-emulsion was administered intra-dermally under isoflurane anesthesia at three sites at the base of the tail, totalling two hundred microliters in volume. Animals were randomized directly thereafter and blindly treated with vehicle (saline, *n* = 15) or liraglutide (200 μg/kg; *n* = 15) s.c. twice-daily. This dose is neuroprotective in mice (DellaValle et al., 2014) and clinically relevant to the anti-diabetic effect in humans (Raun et al., 2007). Healthy controls were treated similarly without EAE emulsion (vehicle, *n* = 7; and liraglutide, *n* = 6).

### Clinical scoring and predefined endpoints

Clinical scoring was performed blinded by two observers twice-daily using the following scale relating to progressive degrees of paralysis: 0, No clinical signs of EAE; 1, Abolished tail tone; 2, Mild paresis of one or both hind legs; 3, Moderate paresis of one or both hind legs; 4, Severe paresis of one or both hind legs; 5, Paresis of one of both hind legs and incipient paresis of one or both forelegs; 6, Moribund. Animals were deemed terminally ill according to predefined humane endpoints designed in consultation with the Danish Animal Inspectorate: animals registering a clinical score of ≥4, a ≥20% loss of initial body weight or when animal caretakers deemed an animal to be moribund before clinical score of 4.

The study was designed to terminate on the peak of disease severity to assess the effect of liraglutide on the acute phase (day 11) before remission. Animals reaching predefined humane endpoints before day 11 were terminated (clinical score of ≥4 or a ≥20% loss of initial body weight).

### Immunoblotting

Brains were removed and the right cerebrum and brainstem were isolated and stored at −80°C (vehicle, *n* = 6; liraglutide, *n* = 7) for immunoblotting. In our previous work in this model, the brainstem shows marked pathological changes in gene expression at day 9 with increased pro-inflammatory and reduced anti-inflammatory cytokines (Pedersen et al., 2013). Brain tissue was homogenized with protease + phosphatase inhibitors (Roche, complete mini; Phosphosafe; Millpore; DK), protein content quantified, aliquoted and stored at −22°C. Thirty micrograms of protein was run on 12% bis-tris gels in MES buffer, transferred to PVDF membranes and blocked in 5% tris-buffered saline + skim milk powder + 0.05% Tween. Primary antibodies were applied in blocking solution: anti-manganese superoxide dismutase (MnSOD), Millipore 06-984, 1:1000; anti-amyloid precursor protein (APP), Abcam 32136, UK, 1:1000; anti-glial fibrillary acidic protein (GFAP), DAKO, IS52430, DK; anti-glyceraldehyde 3-phosphate dehydrogenase (GAPDH), Millipore MAB 374, DK; 1:10,000. Secondary antibodies- anti-rabbit/anti-mouse secondary antibodies (Dako, DK)—were applied 1:2000 and 1:3000, respectively, and visualized with SuperSignal Femto substrate (Thermo Scientific, Denmark) and CCD camera (Bio-Rad Chemidoc XRS imager, Denmark). Images were quantified with ImageJ and reported relative to housekeeping protein GAPDH.

### Data analysis

Clinical scores: Mann–Whitney for individual time points. Cumulative survival: Log-Rank test. Weight: normality (Shapiro–Wilk), thereafter two-way ANOVA and Holm–Sidak multiple comparisons test. Immunoblotting: normality, and Student's *t*-test for parametric data (APP, GFAP, MnSOD_cerebrum_) and for non-parametric data (MnSOD_brainstem_): log-transformation, normality test, and thereafter Student's *t*-test.

## Results

**Figure 1:**
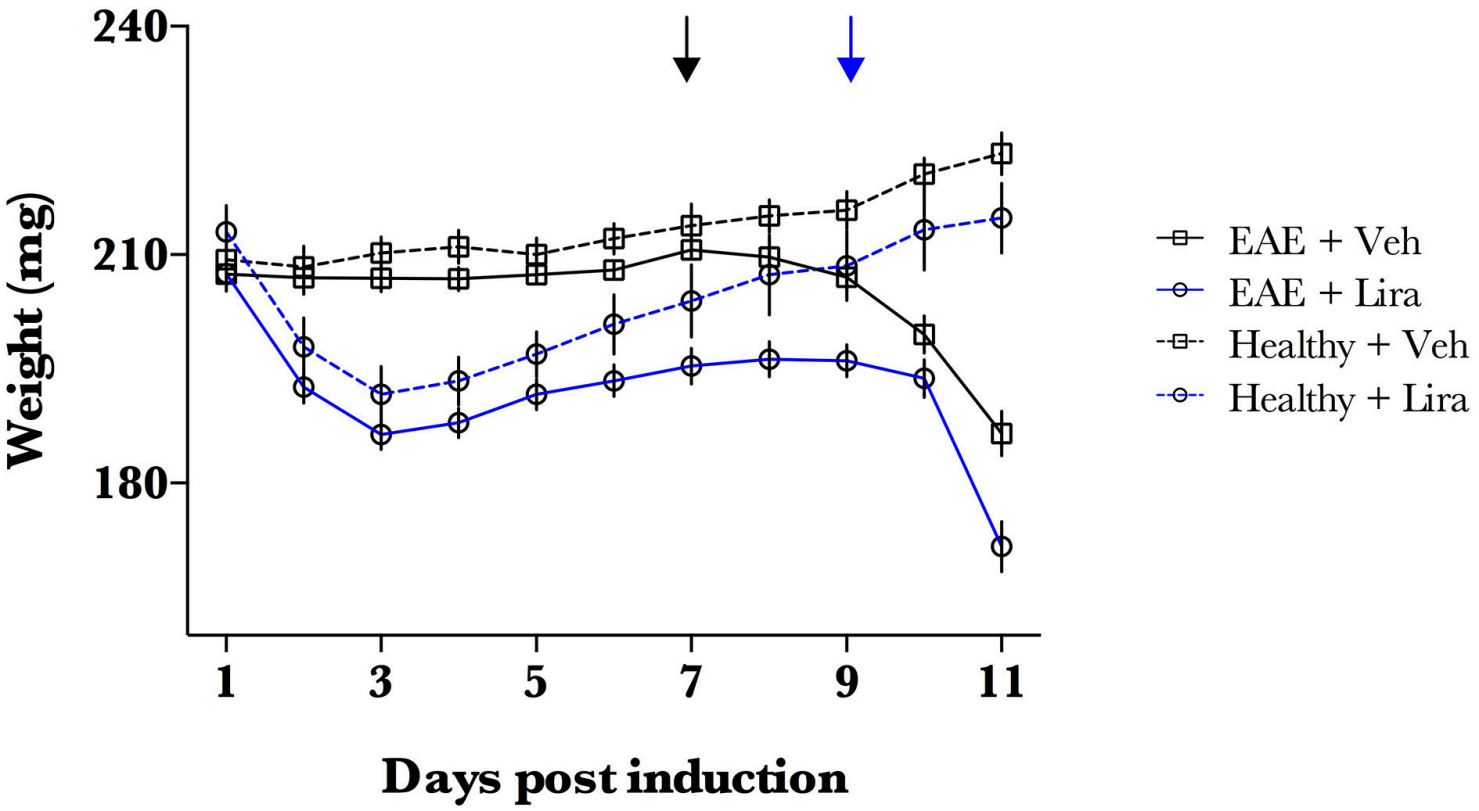
**EAE-emulsion induces weight loss in all animals**. EAE was induced with EAE emulsion at day 0. Animals were randomly selected for vehicle (Veh) and liraglutide (Lira) treatment arms and treated by blinded investigators twice-daily with saline (*n* = 15) or 200 μg/kg of Lira (*n* = 15) (s.c.). Healthy animals were treated equally without EAE emulsion (*n* = Veh:7, Lira:6). Weight of EAE (closed line) and healthy (dotted line) animals for each treatment arm (Veh:black; Lira:blue). Lira treatment induces weight loss at the initial phase of the experiment. All animals receiving EAE-emulsion experienced a weight loss, even in animals with clinical score of 0 at day 11. This reflects a full penetrance of the induction. Arrows denote the interval when mean weight loss began for Veh (black) and Lira (blue) and is described as EAE-associated weight loss phase. Statistics are derived from: normality test (Shapiro–Wilk), thereafter two-way ANOVA and Holm–Sidak multiple comparisons test.

**Figure 2:**
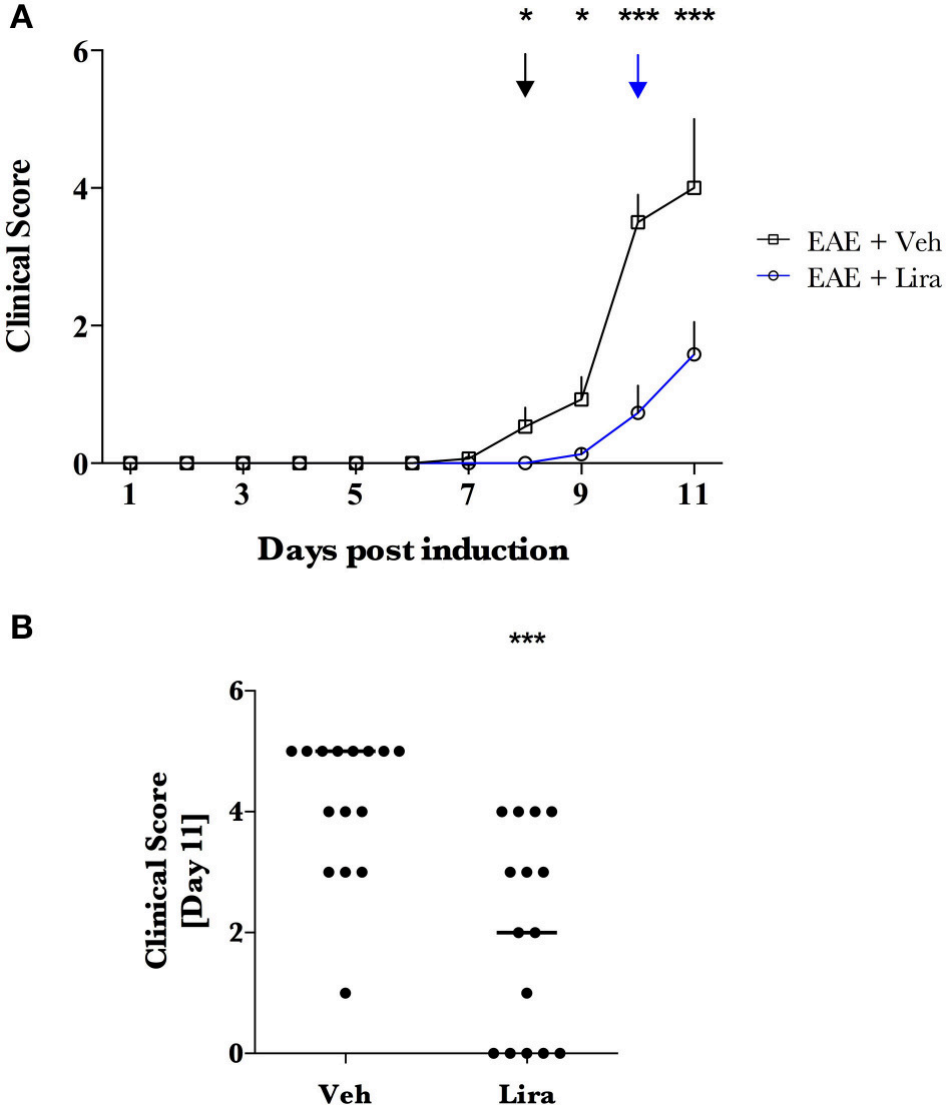
**Liraglutide treatment delays clinical presentation and reduces clinical score in EAE**. EAE was induced with EAE emulsion at day 0. Animals were randomly selected for vehicle (Veh) and liraglutide (Lira) treatment arms and treated by blinded investigators twice-daily with saline (*n* = 15) or 200 μg/kg of Lira (*n* = 15) (s.c.). Healthy animals were treated equally without EAE emulsion (*n* = Veh:7, Lira:6). Clinical scores were conducted twice-daily **(A)** and plotted as Veh (black) and Lira (blue) median ± interquartile range. *Disease debut*: a groupwise clinical score that was significantly higher than 0 is denoted in **(A)** with arrows for Veh (black) and Lira (blue). Asterisks represent a significant difference in animals with EAE treated with Veh vs. Lira. **(B)** Median clinical score at termination (*via* humane endpoint or day 11) is significantly lower in Lira animals than Veh with EAE. Statistics are derived from: **(A,B)** non-parametric analysis of clinical scoring. Statistical significance is reported as ^*^p < 0.05, ^***^p < 0.001.

The penetrance of the EAE induction (defined as a clinical score >0 or EAE-induced weight-loss) was 100% for all rats (Figures 1, 2) and was similar to our previous work in this model (Pedersen et al., 2012). Liraglutide induced weight loss in all animals in the initial days of the study (Figure 1). Weight of healthy liraglutide-treated animals was indistinguishable from healthy-vehicle weight by day 7 (*p* > 0.05).

### Liraglutide delays disease onset and disease progression in EAE

EAE-associated weight loss—described as a downward slope in mean weight- began at day 7 in EAE-vehicle rats and day 9 in EAE-liraglutide rats, pre-empting the presence of clinical symptoms (Figure 1). The disease onset (i.e., first median clinical score statistically significantly >0, Figure 2A) was delayed by liraglutide treatment: day 8 for vehicle—(*p* < 0.05) and day 10 for liraglutide-treated rats (*p* < 0.05). Moreover, EAE-vehicle rats were significantly more impaired than EAE-liraglutide rats at day 8, 9 (*p* < 0.05), and 10, 11 (*p* < 0.0001; Figure 2A). The clinical score at study termination (*via* humane endpoint or day 11) was significantly lower for liraglutide-treated animals: median of 2 vs. 5 (*p* = 0.0003, Figure 2B), where 14 of 15 EAE-vehicle rats achieved the humane endpoint compared to 5 of 15 EAE-liraglutide rats (93 vs. 33%, *p* = 0.0004).

### Liraglutide treatment increases anti-oxidant MnSOD levels and reduces APP

**Figure 3:**
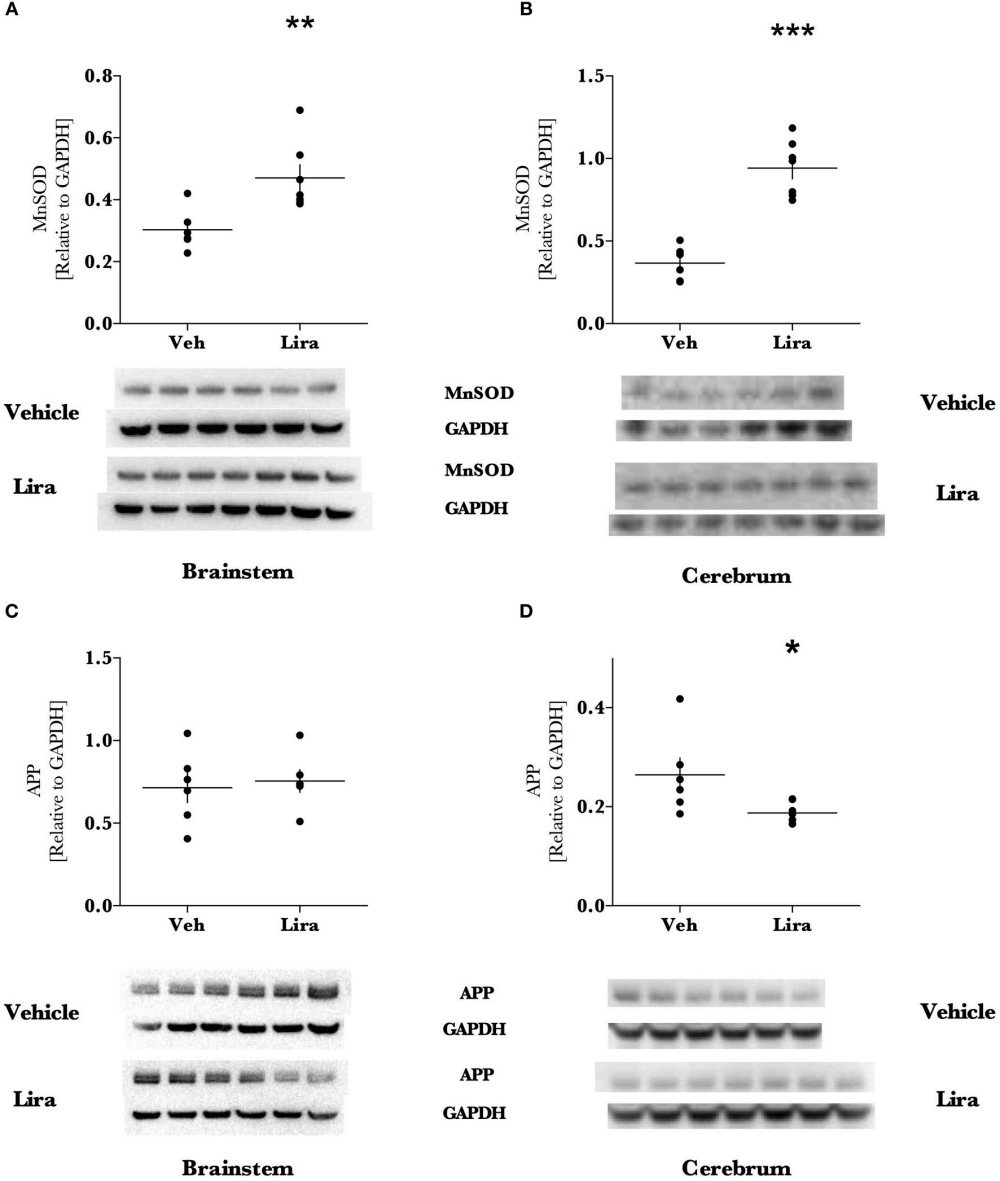
**Liraglutide treatment significantly increases antioxidant capacity and reduces neurodegenerative precursor APP in the EAE brain**. Brains were isolated from EAE animals and the brainstem and right hemisphere of the cerebrum were homogenized for immunoblotting. Manganese superoxide dismutase (MnSOD) levels were significantly increased ~1.6- and 2.6-fold of EAE animals treated with Lira in the brainstem and cerebrum, respectively. Levels of the marker of axonal damage, amyloid-precursor protein (APP), were reduced by 30% in the cerebrum of Lira-treated animals. There was no difference in APP levels in the brainstem of Lira-treated rats. APP was significantly higher in the brainstem than in the cerebrum. All data points are reported as dot plot of MnSOD, and APP levels relative to housekeeping protein GAPDH and significance was tested with parametric analysis after normality was tested (Shapiro-Wilk) **(B–D)**; **(A)** data was log-transformed, re-tested for normality and tested with parametric analysis. Statistical significance is reported as ^*^p < 0.05, ^**^p < 0.01, ^***^p < 0.001; *n* = 6–7.

**Figure 4:**
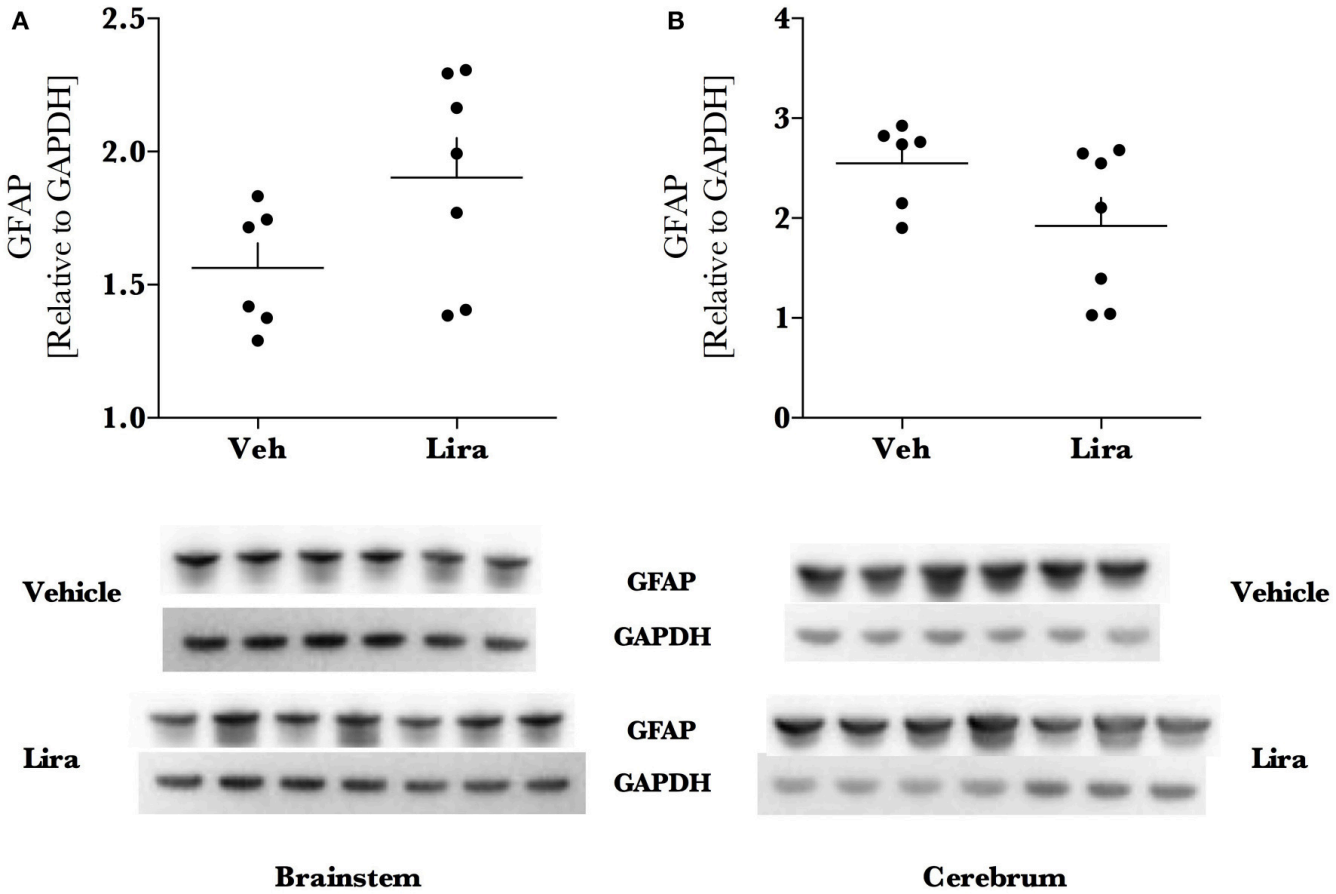
**Liraglutide treatment does not affect astroglial GFAP levels in EAE brain**. Brains were isolated from EAE animals and the brainstem and right hemisphere of the cerebrum were homogenized for immunoblotting. Astroglial marker glial fibrillary acidic protein (GFAP) levels **(A,B)** were significantly higher in the cerebrum than the brainstem but were not affected by liraglutide treatment. All data points are reported as dot plot of GFAP levels relative to housekeeping protein GAPDH and significance was tested with parametric analysis after normality was tested (Shapiro–Wilk); *n* = 6–7.

Liraglutide increased the mitochondrial anti-oxidant protein MnSOD by ~1.6- (brainstem, *p* = 0.003) and ~2.6-fold (cerebrum, *p* < 0.0001) in liraglutide-treated animals relative to the EAE-vehicle group (Figures 3A,B). Liraglutide decreased the neurodegenerative precursor APP (Figures 3C,D) in the cerebrum by 30% (*p* = 0.036) relative to EAE-vehicle rats. APP was significantly higher in the brainstem than the cerebrum (*p* < 0.001) however, was not affected by treatment (*p* = 0.82). GFAP levels were not changed by liraglutide treatment in the respective brain regions (Figures 4A,B). GFAP levels were significantly higher in the cerebrum than the brainstem (*p* < 0.001, Figure 4).

## Discussion

This is, to our knowledge, the first investigation of GLP-1-class agents in an experimental model of MS. Liraglutide reduces clinical debut and severity in this aggressive monophasic model. We look to expand on this clinical effect on the induction phase with further preclinical analysis of different phases of the MS spectrum through various EAE model systems.

Previous work has shown that metformin is effective in EAE (Nath et al., 2009) and thereafter, in MS patients (Negrotto et al., 2016) suggesting that the positive results in this EAE model could be translated into a therapeutic option for MS. Indeed, Negrotto et al. (2016) proposed that weight-loss delays MS development and thus, the weight-reducing capacity of GLP-1 analogs (Candeias et al., 2015) may be beneficial. Additionally, GLP-1 analog treatment is known reduce the adipocyte hormone leptin (Larsen et al., 2001; Iepsen et al., 2015), a pro-inflammatory hormone that is elevated in MS patients and in EAE where a reduction in leptin is protective (Matarese et al., 2010).

Although, these metabolic effects of liraglutide may contribute to the effect of liraglutide described, liraglutide may also activate neuroprotective pathways (Candeias et al., 2015) such as the MnSOD-regulating CREB pathway (DellaValle et al., 2014). Indeed, the increased MnSOD levels (Figure 2) observed in this study supports an improved mitochondrial antioxidant capacity that may play a role in buffering oxidative stress. Mitochondrial dysfunction and frank oxidative damage are present in the brain in EAE (Hasseldam et al., 2016) and previous work in this model has shown that gene expression of the MnSOD-encoding gene, *Sod-2*, is increased in the brainstem in Lewis rats with EAE (Pedersen et al., 2013). These data suggest that this increase in MnSOD is further potentiated with liraglutide treatment. Moreover, we have previously shown that liraglutide reduces reactive oxygen species after traumatic brain injury, preserves mitochondrial function, and is associated with increasing the CREB-regulated, antioxidants: peroxisome proliferator-activated receptor-gamma coactivator-1 alpha and neuroglobin (DellaValle et al., 2014). In recent work on liraglutide treatment in experimental cerebral malaria—a neuropathology with preserved mitochondrial function and without frank reactive oxygen species damage—liraglutide is not protective and does not activate the CREB system (DellaValle et al., 2016). This pre-clinical work suggests that increased antioxidant capacity is an important mechanism of liraglutide-driven neuroprotection. The increased MnSOD levels further support an increased antioxidant capacity at the mitochondrial level.

The pathophysiological role of APP in MS is complex but APP is a marker of cerebral lesions and a biomarker of disease progression and axonal damage in MS and EAE (Matías-Guiu et al., 2016). Recent genomic work suggests that APP may primarily be a marker of early neuronal stress in EAE (Herold et al., 2015). Thus, the reduction in APP observed in the cerebrum (where pathology is diffuse) may reflect a protective mechanism engaged by liraglutide treatment as the pathology develops and/or a reduction in peripheral and central nervous system inflammation in liraglutide-treated animals. Increased MnSOD levels may play a role in controlling buffering these stress signals. Liraglutide did not however affect APP in the brainstem, where- as expected based on the caudal-rostral nature of this model- the APP levels were significantly higher than the cerebrum. This likely reflects increased neuronal stress in the brainstem. This is supported by previous work in the brainstem of this model describing increased gene expression of pro-inflammatory cytokines, reduced anti-inflammatory cytokines yet no signs of cell death signaling (Pedersen et al., 2013).

Liraglutide treatment did not change GFAP expression in the brain although there tended to be an increase in the brainstem of treated animals. Astroglial up-regulation of GLP-1 receptors may be an important mechanism of action for GLP-1 agonism in the brain as GLP-1 receptors are up-regulated in reactive astroglia after cortical lesion (Chowen et al., 1999) and protect astrocytes in culture (Bao et al., 2015). Furthermore, reactive astroglia express neuroglobin in murine EAE (DellaValle et al., 2010), a neuroprotective protein that is up-regulated by liraglutide treatment (DellaValle et al., 2014). We look explore this mechanism further in future studies.

In this investigation we show a strong positive effect of a drug that is already approved for human use, with a well-described safety profile and good tolerance, apart from initial gastrointestinal side effects (Gejl et al., 2016). We demonstrate for the first time that liraglutide delays clinical disease progression in EAE. Moreover, this is associated with improved antioxidant capacity and reduced neuronal damage. Taken together with the promising results in Alzheimer's patients (Gejl et al., 2016), and the prevalence of metabolic disturbances in patients with MS, these data warrant further studies into GLP-1-based therapy as a future contributor to the MS treatment paradigm.

## Author contributions

Substantial contributions to the conception, design of the work (BD, GB, BB, MG, AM, JR, AL); the acquisition, analysis, and interpretation of data for the work (BD, GB, AML, JR, AL); Drafted the work (BD, GB, AL) and revising it critically for important intellectual content (BD, GB, BB, MG, AML, AM, JR, AL); Final approval of the version to be published (BD, GB, BB, MG, AML, AM, JR, AL); Agreement to be accountable for all aspects of the work in ensuring that questions related to the accuracy or integrity of any part of the work are appropriately investigated and resolved (BD, GB, BB, MG, AML, AM, JR, AL).

## Funding

The authors thank A.P. Møller foundation for the financial support.

### Conflict of interest statement

The work of this paper has been performed as independent scientific work with no interferences from any third party. Aarhus University has filed a patent in relation to the findings (Inventor: AL). The other authors declare that the research was conducted in the absence of any commercial or financial relationships that could be construed as a potential conflict of interest.

## Abbreviations

APP: Amyloid precursor protein
DK: Denmark
EAE: Experimental autoimmune encephalitis
GAPDH: Glyceraldehyde 3-phosphate dehydrogenase
GLP-1: Glucagon-like peptide-1
GFAP: Glial fibrillary acidic protein
MnSOD: Manganese superoxide dismutase
MS: Multiple Sclerosis.
